# Psychometric Evaluation of the Chinese Version of the Quality-of-Life Questionnaire for Patients with Multiple Myeloma

**DOI:** 10.1089/pmr.2024.0086

**Published:** 2025-03-03

**Authors:** Chao Liu, Li Wang, Yin Wu, Qian Jiao, Bianhong Yang, Yuan Jian

**Affiliations:** Department of Hematology, Myeloma Research Center of Beijing, Beijing Chaoyang Hospital, Capital Medical University, Beijing, China.

**Keywords:** Chinese version, EORTC QLQ-MY20, multiple myeloma, quality of life, validation

## Abstract

**Background:**

Patients with multiple myeloma (MM) frequently endure a range of disease-related and treatment-related symptoms that significantly impact their quality of life. The Quality of Life Questionnaire Multiple Myeloma Module 20-item (QLQ-MY20) questionnaire developed by European Organization for Research and Treatment of Cancer (EORTC) was to evaluate the quality of life in patients with myeloma. However, it had not been evaluated in China. This study aimed to validate the Chinese version of the EORTC QLQ-MY20 questionnaire in terms of its reliability, validity, and sensitivity.

**Methods:**

The EORTC QLQ-MY20 questionnaire was translated into Chinese and was completed by 140 Chinese patients with MM. The analysis included assessing content validity using the scale-level content validity index, evaluating construct validity through exploratory factor analysis, and calculating reliability statistics.

**Results:**

The scale-level content validity index/unanimity was 0.875, and the scale-level content validity index/average was 0.915. Through exploratory factor analysis, the Chinese version of QLQ-MY20 demonstrated a test-retest reliability of 0.857. The Cronbach’s α coefficient was 0.887, with the three dimensions having coefficients of 0.879, 0.809, and 0.786, respectively. The Kaiser–Meyer–Oklin value was 0.927, and the eigenvalues for the three common factors were 10.127, 2.831, and 1.124, respectively. The overall variance contribution rate was 74.112%.

**Conclusions:**

The Chinese edition of the EORTC QLQ-MY20 is deemed a reliable and valid instrument for evaluating the quality of life in Chinese patients with MM.

## Introduction

Multiple myeloma (MM) is defined by the clonal expansion of plasma cells in the bone marrow.^1^ It represents about 13% of hematologic malignancies and accounts for 20% of related deaths.^2,3^ In China, MM represented the third most prevalent hematological malignancy, with approximately 14,000 new diagnoses each year, corresponding to an incidence rate of approximately 1 per 100,000.^4^ Due to the common side effects of induction therapy and hematopoietic stem cell transplantation, patients with MM frequently suffer from a significant symptom load, either due to the disease itself or as a result of its treatment, including experiencing bone pain, muscle weakness, fatigue, vomiting, nausea, constipation, diarrhea, and peripheral neuritis.^1,5^ The U.S. Food and Drug Administration has identified both the improvement of survival rates and the enhancement of the quality of life as critical factors for assessing the effectiveness of cancer therapies.^6^ Consequently, there is a pressing requirement to understand the quality of life of patients with MM in China. Health-related quality of life (HRQoL) represents a crucial endpoint in MM research, offering essential information to support treatment decision making for physicians and patients.^7,8^ The HRQoL questionnaire that is most widely utilized in MM research is the Quality of Life Questionnaire-Core 30 (QLQ-C30) developed by the European Organization for Research and Treatment of Cancer (EORTC).^9^ However, while the QLQ-C30 captures general aspects of HRQoL, it does not comprehensively address MM-specific concerns.^10^

For this reason, the EORTC developed a supplemental, myeloma-specific module, termed Quality of Life Questionnaire-Multiple Myeloma 20 (QLQ-MY20). The QLQ-MY20 has shown reliability and validity in assessing the quality of life for patients with MM.^11–14^ Nonetheless, the QLQ-MY20 has not been utilized or evaluated in China to date. Therefore, we conducted this study and aimed to translate the QLQ-MY20 into Chinese and to evaluate its properties in the Chinese population psychometrically.

## Participants and Methods

### Participants

Participants for this study were recruited from September 2018 to March 2019 in the Department of Hematology, Beijing Chaoyang Hospital, Capital Medical University. The inclusion criteria were as follows: patient ≥18 years old and should meet the diagnostic criteria of MM according to the 2014 International Myeloma Working Group criteria,^15^ with simplified Chinese reading and writing ability and normal language expression ability. All patients had signed the consent forms and volunteered to participate in this study. Exclusion criteria included cognitive impairment, mental illness, other life-threatening severe diseases or complications, or unwillingness to participate.

### EORTC QLQ-MY20

The EORTC QLQ-MY20 includes 20 questions, organized into three main scales, future perspective (3 items), disease symptoms (6 items), and side effects of treatment (10 items), along with 1 item on body image. Participants rated each item on a scale from 1 to 4, with “not at all” (1) and “very much” (4) as the anchors. Raw scores were then linearly converted to a 0–100 scale,^11^ where higher scores denote either better health status or more severe symptoms.

The EORTC QLQ-MY20 questionnaire effectively captures how the disease duration and the effects of treatment influence patients’ quality of life. Therefore, additional options to assess these factors separately were not necessary. The main treatments included bortezomib, liposomal doxorubicin, dexamethasone, and others, while the main side effects encompassed neuritis, cytopenia, infection, hypertension, hyperglycemia, and gastrointestinal symptoms, which were already covered in the questionnaire items. Consequently, there was no need for additional options to address these specific concerns.

### Translation procedure

The Chinese version of the EORTC QLQ-MY20 scale underwent a meticulous translation process to ensure an accurate representation of the original scale. This process included: (1) Literal translation: It was conducted by two individuals: one hematology doctor and one nurse, both of whose native language was Chinese, using simple language to accurately express the content of the scale. The two translators independently prepared the first and second drafts of the translation, which were then reviewed and refined by a third-party nursing graduate student whose native language was also Chinese. Each item was selected as a more appropriate translation version. These items were compiled to form the third draft. (2) Back-translation: The third draft was back-translated into English by a researcher familiar with the research domain but unfamiliar with the original English scale. The back-translated version was reviewed by professionals to ensure that it aligned with the original scale’s intent. Then the simplified Chinese version of the EORTC QLQ-MY20 scale was finally determined.

### Cultural debugging

The study invited six experts to appraise the Chinese version of the EORTC QLQ-MY20 scale. The six experts include three hematology clinicians, two hematology clinical nurses, and one hematology nursing manager. Drawing on their professional theoretical expertise and clinical experience, these experts were tasked with assessing the scale using a 4-point Likert scale, where 1 indicated “not relevant,” 2 indicated “partially relevant,” 3 indicated “relevant,” and 4 indicated “very relevant.” Following their review, adjustments were made to 5 out of the 20 items related to the scale. For example: “If you had pain did it increase with activity?” is affected by Chinese language habits, this item should be translated to “如果您有过疼痛, 疼痛是否随着活动而加重?” instead of “如果您有过疼痛, 疼痛是否随着活动而增加?”; “Did you feel drowsy?” should be correctly translated to “您有过昏昏欲睡的感觉吗?” instead of “您昏昏欲睡吗?”

### Pretesting

To gauge the understandability and acceptability of the translated scale, a pretest was conducted. Ten patients with MM who met specific inclusion criteria were pretested in the Department of Hematology, Beijing Chaoyang Hospital, Capital Medical University. Patients were asked to complete the scale on their own, with the option to dictate their responses if they were unable to write. After completing the scale, the investigator interviewed 15 subjects and asked them how difficult it was to understand and answer these scale items. The consistency of opinions among the sample population should be at least 80%. If 20% or more of the patients found certain items unclear, these items were to be adjusted until the clarity of these items is above 80%. The principle of adjusting items should be based on the balance among the Chinese meaning of English vocabulary, context, brevity, understandability, and Chinese language habits. After carrying out the processes mentioned above, the simplified Chinese version of the scale was finally completed. According to the feedback of the research subjects, the items of this scale are easy to understand and related to the disease.

### Sample size calculation

According to the literature,^16^ the sample size of the reliability and validity test of the questionnaire should be 5–10 times the number of items. In this study, an intermediate ratio of 1:7.5 was chosen. Since a total of this questionnaire with 20 items, the total sample size should be 150 cases. In this study, eligible patients with MM who fulfilled the inclusion criteria were asked to fill out the questionnaire based on informed consent. The investigators used standardized instruction before the questionnaires were issued.

### Statistical analyses

Analyses were carried out using IBM SPSS Statistic 23.0. Pearson correlation coefficient was employed to calculate the test-retest reliability to check the stability of the scale. The intraclass correlation coefficient (ICC) was used to assess the test-retest reliability. Finally, Cronbach’s α coefficient was calculated to evaluate the internal consistency of the scale.

### Content validity

The individual items of the scale were evaluated by four staff members in related fields (two senior clinical nursing administrators and two senior clinical physicians) based on four methods of division: 1 = no correlation, 2 = weak correlation, 3 = comparison, 4 = strong correlation. This study used the scale-level content validity index (S-CVI) to assess the content validity of the scale. S-CVI measurement uses the same consistent S-CVI (S-CVI/unanimity, S-CVI/UA) and average S-CVI (S-CVI/Ave).^17^ S-CVI/UA refers to the percentage of items that are rated 3 or 4 by all experts as a percentage of all items, indicating the fact that all experts agree that the relevant situation, an S-CVI/UA ≥0.8 prompts content validity is good.^18^ S-CVI/Ave refers to the mean of the ratio of items for each expert rating of 3 or 4 and S-CVI/Ave should be 0.9.^19^

### Structural validity

Exploratory factor analysis was conducted to assess and interpret the structural validity of the scale. The principal component analysis method was applied for the extraction of factors. The orthogonal rotation method was employed to comprehensively estimate the rationality of the factor load. Items with low factor loadings, which indicated a weak association with other items, were considered for removal. The goal of all of them is to obtain the most suitable structure.

## Results

### Patient characteristics

A total of 150 copies were issued, and 140 completed scales were received. The effective rate of recovery was 93.33%. The demographic and clinical characteristics of the participants are detailed in Table 1, including 105 (75%) newly diagnosed patients with MM and 35 (25%) patients with relapsed/refractory MM. The median age for the participants (*n* = 140) was 60 years (range: 42–79).

**Table 1.** Characteristics of the Subjects

	*N* = 140
Age (years)	60 (42–79)
Gender	
Male	78 (55.7%)
Female	62 (44.3%)
DS stage	
I	9 (6.4%)
II	19 (13.6%)
III	112 (80%)
ISS stage	
I	35 (15%)
II	21 (25%)
III	84 (60%)
Disease status	
Newly diagnosed	105 (75%)
Relapsed/Refractory	35 (25%)

Data are presented as *n* (%) or median (range). DS: Durie-Salmon; ISS: International Staging System.

### Test-retest reliability

The ICC was employed to assess the stability of the scale’s scores by utilizing data from a subsample of 40 participants who completed the questionnaire twice through the convenient sampling method, 2 weeks apart. The correlation coefficient ICC value was 0.857 (*n* = 40, *p* < 0.01) using Pearson twice to measure the score and make the retest reliability inspection. All four scales of the questionnaire demonstrated test-retest reliability of 0.8 or higher. The disease symptoms scale exhibited the highest test-retest reliability (ICC = 0.92), confirming the scale’s robust test-retest reliability. The S-CVI/UA of the questionnaire was 0.875, and the S-CVI/Ave was 0.915.

### Construct validity

The size of the Kaiser–Meyer–Oklin measure of sampling adequacy was 0.927, indicating that the items shared sufficient common variance to proceed with factor analysis. Additionally, the significance of Bartlett’s test of sphericity was *χ*^2^ (190) = 2756.069 (*p* < 0.01), which indicated that there were common factors among the matrices of the parent population and it was suitable for factor analysis. The analysis revealed a structure consisting of three factors, which were subsequently named as follows:

1. Emotion (item 16, 20, 19, 15, 18, 12, 13, 14, 11; e.g. “Have you had burning or sore eyes?” and “Have you worried about your health in the future?”).
2. Pain (items 2, 3, 1, 5, 6, 4; e.g. “Have you had pain in your back?” and “Have you had pain in your hip?”).
3. Symptoms and feelings (items 8, 10, 9, 7; e.g. “Did you feel thirsty?” and “Have you had a dry mouth?”).

The rotated factor values ranged from 0.547 to 0.884, as detailed in Table 2. Items with factor loadings below 0.3 were not included in the reporting. In cases where an item loaded onto more than one factor, the higher loading was taken into account, with the exception of item 17. The three factors accounted for 74.112% of the total variance, as shown in Table 3. The outcomes of the item loadings for each factor, the eigenvalues, and the percentage of variance explained by each factor corroborate the three-factor structure. The eigenvalues, the percentages of variance explained, and Cronbach’s α coefficients are presented in Table 3.

**Table 2.** Rotating Component Matrix

Item	Factor 1	Factor 2	Factor 3
16. Have you had burning or sore eyes?	0.884	0.168	0.268
20. Have you worried about your health in the future?	0.879	0.147	0.251
19. Have you been worried about dying?	0.871	0.076	0.259
15. Have you had acid indigestion or heartburn?	0.83	0.128	0.353
18. Have you been thinking about your illness?	0.83	0.128	0.353
12. Answer this question only if you lost any hair: Were you upset by the loss of your hair?	0.82	0.122	0.29
13. Did you have tingling hands or feet?	0.811	0.193	0.165
14. Did you feel restless or agitated?	0.763	0.37	0.26
11. Have you lost any hair?	0.715	0.036	0.279
2. Have you had pain in your back?	−0.006	0.848	0.152
3. Have you had pain in your hip?	0.174	0.825	0.072
1. Have you had bone aches or pain?	−0.178	0.815	0.214
5. Have you had pain in your chest?	0.376	0.7	0.1
6. If you had pain, did it increase with activity?	0.335	0.691	0.22
4. Have you had pain in your arm or shoulder?	0.291	0.67	0.036
8. Did you feel thirsty?	0.335	0.149	0.826
10. Have you had a dry mouth?	0.444	0.178	0.796
9. Have you felt ill?	0.406	0.237	0.712
7. Did you feel drowsy?	0.46	0.263	0.547

**Table 3.** Eigenvalues, Percentages of Variance, and Cronbach’s **α** (Reliability)

OLO-MY20 factors	Eigenvalue (*N* = 140)	%Variance (*N* = 140)	Cronbach's **α** (*N* = 140)
1. Emotion	10.127	77.134	0.879
2. Pain	2.831	72.786	0.809
3. Symptoms and feelings	1.124	73.453	0.786
Total		74.112	0.887

After carrying out principal component analysis, each item meets the condition that there is a higher load on a particular common factor and a lower load value on other common factors. Three common factors were obtained by orthogonal rotation by using the maximum difference method. The characteristic values were 10.127, 2.831, and 1.124, respectively. The cumulative contribution rate of variance was 74.112%. After rotating by the maximum variance orthogonal rotation method, all item factor loads are higher than 0.5. The composition matrix behind the rotating shaft is shown in Table 2.

The Cronbach’s α of the total questionnaire was 0.887, which is higher than 0.80 and three multi-item scales (0.879, 0.809, 0.786), indicating that the scale has good internal consistency.

## Discussion

Patients with MM commonly suffer from a variety of symptoms due to the disease, including fatigue, hypercalcemia, and bone pain.^20^ After diagnosis, patients with MM often begin chemotherapy, which can result in significant side effects such as poor mental state, gastrointestinal reactions, and peripheral neuropathy, profoundly affecting their quality of life.^21^ Quantitative identification of patients’ quality of life is important to implement early intervention and to improve clinical outcomes.^22^ K. Cocks’s research has underscored the utility of the QLQ-MY20 in assessing quality of life in patients with MM, capturing additional aspects such as body image and future perspectives across seven countries.^11^

In our study, the Chinese version of the QLQ-MY20 demonstrated adequate construct validity and reliability, with excellent test-retest reliability indicating its acceptability among patients. To the best of our knowledge, this study represents the first instance of testing the reliability and validity of the QLQ-MY20 scale in China. The Cronbach’s α coefficients for each factor within the total scale ranged from 0.786 to 0.879, with an overall alpha coefficient of 0.887, suggesting good internal consistency reliability for the Chinese version of the EORTC QLQ-MY20. These alpha coefficients were consistent with those reported for the original English and Greek versions^11,13^ and higher than those observed in the Mexican–Spanish version.^12^ We also assessed the test-retest reliability of the Chinese QLQ-MY20 in a subset of 40 clinically stable patients over a 2-week period, achieving an ICC value of 0.857. This indicates that the Chinese version exhibits excellent retest reliability and time stability in assessing the quality of life of patients with MM.

Factor analysis of the scale revealed three key dimensions, emotion, pain, and symptoms/feelings, which are consistent with the original scale. Our findings emphasize the significance of emotion and pain as key determinants of the EORTC QLQ-MY20 in patients with MM. Higher levels of functioning and less severe symptoms are associated with a better quality of life. The QLQ-MY20 has received comprehensive clinical and psychometric validation, covering all HRQoL issues pertinent to patients with MM.^21^ Studies by Kontodimopoulos and colleagues on the Greek version of the QLQ-MY20 have shown similar psychometric properties to the original English version, which reinforces the consistency of our findings.^13^

The cumulative contribution rate of the factors was 74.112%. The majority of the items loaded as anticipated, and the factor loadings exceeded the minimum recommended threshold of 0.4, in line with the standards suggested by Hair et al.^23^ Generally, a scale is considered to have good structural validity if its common factors can explain over 40% of the variance, with most items loading above 0.3 on their respective factors. The results from our study indicate that the Chinese version of the EORTC QLQ-MY20 exhibits good construct validity, and its three-dimensional structure aligns with statistical requirements, indicating a reasonable scale structure.

Also, this study had some limitations. The limited sample size of our study limited its generalizability. Additionally, as a single-center study, it needs to be validated in a broader scope. Although test-retest reliability and construct validity of the Chinese version of QLQ-MY20 were satisfactorily demonstrated, the scale needs to be tested among patients with diverse backgrounds in various treatment settings.

## Conclusion

In conclusion, our study demonstrates that the Chinese version of the QLQ-MY20 is a feasible, dependable, and valid instrument for evaluating the particular quality of life of patients with MM. This scale can assist clinical staff in understanding the life quality of patients with MM and in actively intervening to enhance their quality of life and prognosis.

## Data Availability

The corresponding author needs to be contacted for data requests.

## Declarations

All procedures performed in studies involving human participants were under the ethical standards of the institutional and/or national research committee and complied with the 1964 Helsinki Declaration and its later amendments or comparable ethical standards.

## Abbreviations Used

DS: Durie-Salmon
EORTC: European Organization for Research and Treatment of Cancer
HRQoL: health-related quality of life
ICC: intraclass correlation coefficient
ISS: International Staging System
MM: multiple myeloma
QLQ-C30: Quality of Life Questionnaire-Core 30
QLQ-MY20: Quality of Life Questionnaire-Multiple Myeloma 20
S-CVI: scale-level content validity index
S-CVI/Ave: scale-level content validity index/average
S-CVI/UA: scale-level content validity index/unanimity
